# Study on Correlation of Mechanical and Thermal Properties of Coal-Based Carbon Foam with the Weight Loss Rate after Oxidation

**DOI:** 10.3390/ma15144887

**Published:** 2022-07-13

**Authors:** Degang Wang, Qikai Zhuang, Kai Li, Yanfei Wang

**Affiliations:** 1 College of Aerospace Science and Engineering, National University of Defense Technology, 109 Deya Road, Changsha 410073, China; darwin_wang@163.com (D.W.); 750455@sohu.com (Y.W.); 2 School of Aeronautic Science and Engineering, Beihang University, Beijing 100191, China; 3 State Key Laboratory of NBC Protection for Civilian, Research Institute of Chemical Defense, Beijing 100191, China; k2005@sdut.edu.cn

**Keywords:** coal-based carbon foam, mechanical properties, thermal properties, oxidation behavior

## Abstract

With the increasing demand for high temperature-resistant heat insulation materials for hypersonic vehicles, carbon foam has been studied extensively, and its mechanical and thermal properties have been fully researched, but the oxidation behavior of carbon foams during service and the change in their properties after oxidation are rarely studied. This paper studied the relationship between both mechanical and thermal properties and oxidation degree of two kinds of foams, coal-based carbon foam and antioxidant coal-based carbon foam with chemical vapor deposition of SiC particles. The variation trend for the two kinds of foam was the same. When the oxidation degree was less than 15%, the compression modulus and strength weakened with the increase in weight loss rate, but the thermal conductivity decreased with the increase in weight loss rate, which was a favorable influence for the thermal protection system. The mechanical and thermal properties had a linear relationship with the weight loss rate, but the slope was different between 0% to 10% and 10% to 15%. The microscopic mechanism of these changes was also analyzed.

## 1. Introduction

As the cruising speed Mach number of hypersonic vehicles increases, the problem of aerodynamic heating becomes more prominent, especially along the sharp parts of the aircraft, where temperature can even exceed 1500 °C [1]. However, currently extensively used thermal insulation materials, such as alumina-enhanced thermal barrier (AETB), low-temperature reusable surface insulation tiles (LRSI), flexible reusable surface insulation (FRSI), etc., are all oxide ceramics, and their long-term use temperature is generally lower than 1200 °C [2]. Limited by available materials, the current thermal protection scheme for hypersonic aircraft in the ultra-high temperature area is active cooling thermal protection, which leads to a high weight cost for the thermal protection system [3,4,5]. Therefore, new materials with high temperature resistance and thermal insulation are urgently needed to enable passive thermal protection to replace active cooling thermal protection, which will make an important contribution to reducing the weight of aircraft. Carbon foam (CF) is a new type of lightweight carbon-based thermal insulation material that can meet the above requirements. It can work at 2400 °C for a long time with effective anti-oxidation measures, and maintain low thermal conductivity at high temperatures [6].

The basic properties of CF have been studied. Janszen [7] studied the relationship between mechanical properties and strain rate of CF under high-speed impact. Grujicic [8] studied the impact resistance of common aero vehicles (CAVs) with carbon–carbon/carbon–foam fuselages. Takuya Aoki [9,10,11] studied the application of the sandwich structure formed by CF and carbon fiber reinforced resin or ceramic matrix composite on the Mars lander. This kind of structure with both bearing capacity and thermal insulation capacity belongs to integrated thermal protection structure (ITPS). Sarzynski [12] investigated the tensile and shear properties and failure modes of resin-based CF through experiments. CF exhibited brittle fractures in both cases, with an average tensile and shear modulus of 0.269 GPa and 0.145 GPa, respectively, and the actual Poisson’s ratio was between −0.3275 and 0.5. The authors did not provide the tensile and shear strength of CF in the paper. Then, the flexural properties of CF-cored carbon fiber/epoxy composite sandwich beams were studied by experimental, analytical and finite element methods. The test results show that the main failure mode of the CF sandwich beam was the shear failure of the core material. The authors preliminarily estimated the material properties using classical laminate theory, treating the sandwich beam as a laminate composed of panels and CFs, and found that the calculation results were not ideal, indicating that classical laminate theory is not suitable for predicting the stress of this material system and geometry and strain field. To this end, the author established a finite element model of the sandwich beam, the foam was treated as a dense isotropic material, and the strain and stress in the bending beam were predicted using a two-parameter progressive damage model. The prediction results of the finite element model were basically consistent with the experimental results. Sihn [13] measured the compressive and shear mechanical properties of CF provided by different manufacturers. The results show that CFs provided by different manufacturers exhibit similar properties, and the properties of CFs along the foaming direction and perpendicular to the foaming direction show significant differences. When placed in air at 316 °C for 100 h, the foam weight loss rate was 0.6%, and the compressive strength and modulus were almost not degraded.

The challenge for CF applications in TPS is oxidation, and even if anti-oxidation measures are taken, this phenomenon can only be delayed rather than completely avoided. Oxidation not only jeopardizes the integrity of the structure, but also changes the properties of the material, resulting in structural properties that do not meet the requirements for use [14,15,16,17,18]. Therefore, the oxidation behavior of CF is one of the important factors affecting the service life of TPS, which is a main indicator in the design of aircraft with high endurance and high Mach number. Quantifying the effect of oxidation on the mechanical and thermal properties of CF is a key factor in determining the service life of CF [19,20,21,22], but there is currently no relevant literature. In this paper, the mechanical properties and thermal properties of CF and antioxidant coal-based carbon foam (ACCF) were studied. The relationship between properties and oxidative weight loss rate was examined.

## 2. Experiment

The CF production process studied in this paper is as follows: pulverize the finely washed bituminous coal with a free expansion number of 5.5 to more than 100 mesh, put it into a mold, put it into a high temperature and high pressure reaction kettle with the mold, seal it and vacuumize it to below 0.001 MPa, close the vacuum, and pass high-purity nitrogen until the pressure reaches 1.5 MPa, heat up to 500 °C at a heating rate of 1 °C/min, keep the constant temperature for 300 min, naturally cool to room temperature, depressurize to atmospheric pressure, open the reactor, take out the material, and put it in a vacuum sintering furnace. Under vacuum conditions, the temperature was raised to 1650 °C at a heating rate of 1 °C/min, kept at a constant temperature for 300 min, and then cooled to room temperature at a cooling rate of 2 °C/min to obtain the CF required for the test [23,24,25]. It is worth noting that bituminous coal contains metallic impurities, which are very persistent and difficult to remove. Even high temperature annealing at 1650 °C at a heating rate of 1 °C/min, for 300 min will not remove the temperature-resistant metallic impurities, nor will acid washing [26]. Such impurities can affect resistance against oxidation, and their effects on carbon foam change with raw materials.

A part of the above-mentioned CF was further anti-oxidative treated, and its process was as follows: CF is put into a chemical vapor deposition furnace, and the temperature is raised to 1075 °C after evacuation. Methyl-trichloro-silane (MTS) was carried by means of H_2_ bubbling in MTS to the reaction chamber, employing H_2_ as the carrier and catalyst gas and argon as the dilute gas. The H_2_ flow rate was kept at 300 mL/min, while the Ar flow rate was kept at 200 mL/min. The deposition time and pressure were 300 min and 1.4 kPa, respectively. Then, the required ACCF was finished [27,28,29].

### 2.1. Specimens

**Figure 1:**
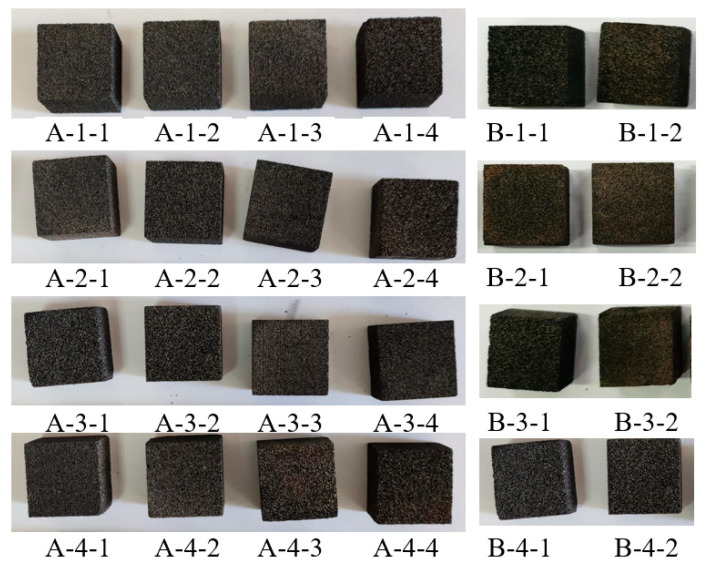
Specimens before experiment.

In order to study the relationship between the mechanical and thermal properties of CF and the oxidative weight loss rate, this paper designed four groups of control tests, with four specimens in each group for a total of 16 specimens, numbered as A-1-1~A-1-4, A-2-1~A-2-4, A-3-1~A-3-4, and A-4-1~A-4-4, respectively. The preset oxidative weight loss rates of each specimen were 0%, 5%, 10% and 15%, respectively. In order to study the relationship between mechanical and thermal properties of ACCF and oxidative weight loss rate, limited by raw materials, this paper designed four groups of control tests, with two specimens in each group for a total of eight specimens, numbered as B-1-1, B-1-2, B-2-1, B-2-2, B-3-1, B-3-2, B-4-1, and B-4-2. The preset oxidative weight loss rates for each specimen were 0%, 5%, 10% and 15%, respectively. The size of the test parts was 30 mm × 30 mm × 15 mm [30,31,32], as shown in Figure 1.

### 2.2. Oxidation Treatment

Specimens are calcined for 30 min in a tubular furnace at 400 °C under flowing nitrogen atmosphere to remove impurities. The specimens are taken out, cooled to room temperature, and weighed to obtain initial mass. The specimens are oxidized to a designed oxidative weight loss rate in a small tubular furnace as follows: (a) The specimen was put into the tubular furnace and heated to 600 °C at 10 °C/min for 5 min; (b) nitrogen at room temperature was fed into the furnace with a flow rate of 0.5 L/min for 3 min, to fully expel the oxidation exhaust gas and cool down the furnace temperature to 360–380 °C; (c) Fresh air at room temperature is fed into the furnace with a flow rate of 0.7 L/min for 5 min; the furnace temperature drops to 230–290 °C; (d) after repeating step A to C several times, take out the specimen and cool it to room temperature for weighing. Stop the test if the preset oxidation weight loss rate is met; otherwise, continue the oxidation.

Step b and step c are the key to ensure the success of the test. In step b, specimens are cooled to below 400 °C, which is the starting temperature of carbon material oxidation, to avoid allowing the subsequent air into the outer surface at the beginning of oxidation. In step c, the tubular furnace should be kept heating while the air is passing through, which enables fresh air to get into the pores sufficiently. This will ensure the uniformity of the oxidation degree inside and outside the material.

### 2.3. Thermal Properties Tests

After the oxidation treatment of the specimens, the thermal property test is carried out first, because the thermal property test process will not damage the test part, and the test part can still be used for mechanical property measurement. In this paper, Hot Disk-TPS2500s (ISO 22007-2) transient heat flow method was used to test the thermal performance of specimens. The equipment requires that the specimens clamped with the probe have the same or similar thermal performance. The probe has a heating function and calculates the average thermal conductivity of the two specimens by measuring the heat flow on the back of the specimens. The thermal properties of each set of specimens were measured at room temperature (20 °C).

### 2.4. Mechanical Properties Tests

CCF in thermal protection structure is subjected to a complex stress environment, and the basic mechanical properties of CCF include tensile, compression and shear properties. This paper only studied the compression properties of CF, because the compression properties of CCF were obtained by flat pressure test (ASTM C365/C365M), which is the simplest test method and consumes less material. Moreover, for porous materials such as CCF, their tensile, compressive and shear properties are all related to the mechanical properties of the carbon skeleton, and the relationship between all of the mechanical properties of the carbon skeleton and oxidative weight loss rate can be well revealed by studying the compressive properties. The flat pressure test was carried out on an Instron-8802 electro-hydraulic servo material test machine with loading speed of 1 mm/min.

### 2.5. Scanning Electron Microscopy

Since CCF is a brittle material, the unbroken part far from the fracture still has a relatively complete structure after mechanical property test, and it can be made into a SEM sample to observe the oxidation morphology of the pore wall. The model of SEM device is a TESCAN MIRA, at an accelerating voltage of 20 kV.

## 3. Discussion

### 3.1. Results

Mechanical and thermal experiment results are listed in Table 1. As it is difficult to continuously monitor the quality changing process of the specimens, the actual value of the oxidation weight loss rate of the specimen is slightly different from the designed, but this does not affect the subsequent data analysis.

### 3.2. Mechanical Properties Analysis

**Figure 2:**
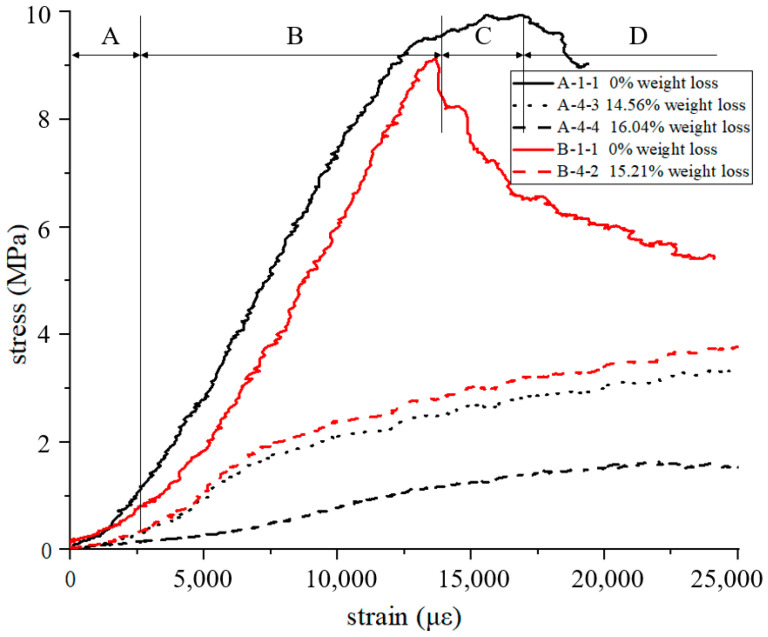
Stress-strain curve of A-1-1, A-4-3, A-4-4, B-1-1, B-4-2.

Due to the large amount of data, the stress–strain curve was drawn from the data of the two specimens with the largest and smallest oxidation weight loss rates in CCF and ACCF, as shown in Figure 2. A-1-1 is the uniaxial compressive stress-strain curve of the original CCF without oxidation weight loss. The curves show typical brittle foam compression behaviors that are divided into 4 regions, defined as regions A to D:

- In region A, different areas of the specimen participate in the bearing successively at the beginning of the test, causing the slope of the curve to increase, and the surface of the specimen is completely compacted with the platen in the end;
- In region B, CCF is uniformly stressed and linear, which is the main working section of the CCF. The slope and the max stress of region B are the compression modulus and compression linear strength of CCF, which are the most concerned performance data in the design of thermal protection structures, as listed in Table 1;
- In region C, the slope decreases to minus, which means failure occurs and extends. At the end of region C, the foam is completely destroyed in the section perpendicular to the load direction, and the material should not be allowed to work at this stage when designing the structure;
- In region D, the CCF continues to failure, and fragments into several pieces in the longitudinal direction. This region is short, which cannot show the long platform area of plastic foam. This brittle feature is unique to CCF, which contains a large amount of disordered amorphous carbon [28,29,30].

The modulus and strength of A-4-4 are 10.08% and 9.9% of those of A-1-1, respectively, which means the mechanical properties are greatly attenuated. A-4-4 is the uniaxial compressive stress–strain curve of the original CCF after oxidation weight loss of 16.04%. Compared with the curve of A-1-1, it is smoother and lacks an obvious load drop. A-4-4 also did not fracture longitudinally, but was compacted and exhibited plastic foam characteristics. In previous studies [28], it was found that the stress–strain curves of pitch-based carbon foam (PCF) with a very high degree of graphitization were similar to those of A-4-4, showing good plastic properties, which is due to the existence of graphite sheets that are easier to slide relative to each other.

B-1-1 and B-4-2 are the stress–strain curves of ACCF, and it can be found that except for the slightly different modulus and strength (discussed later), they still follow the above rules of CCF.

**Figure 3:**
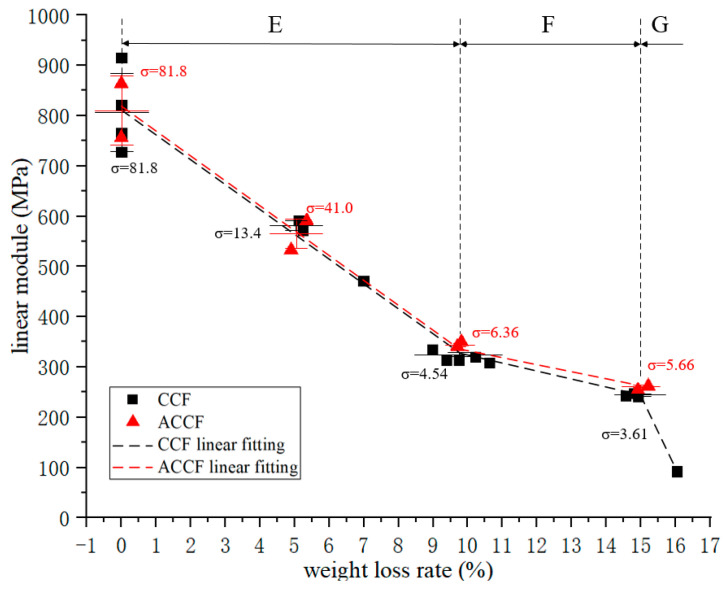
Compression modulus–weight loss rate curve.

The compressive modulus–oxidative weight loss rate curves of all specimens are shown in Figure 3. Overall, the compressive modulus decreases sharply with the increase in the oxidation weight loss rate. The decay curve of CCF is divided into three regions:

- In region E, the compressive modulus decays linearly with the oxidation weight loss rate, and this process continues until the CCF loses 10% of its weight, at which time the compressive modulus is about 40% of the initial value;
- In region F, the rate of decay of the compressive modulus is significantly reduced;
- In region G, it shows that when the weight loss rate of CCF oxidation exceeds 15%, the compressive modulus decays faster than that in region E.

**Figure 4:**
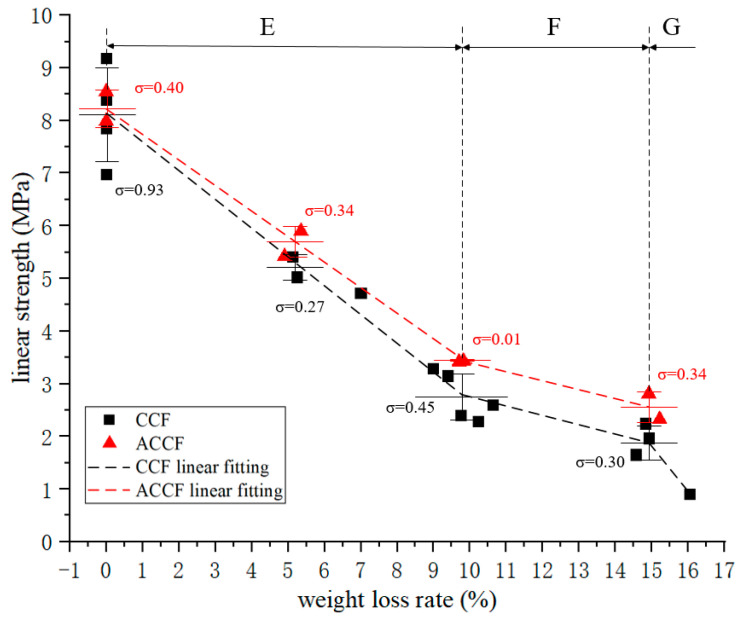
Linear compression strength–weight loss rate curve.

The compressive linear strength–oxidative weight loss rate curve is shown in Figure 4, and its change trend is exactly the same as that of the compressive modulus. It is divided into three obvious regions, and the positions of the turning points are the same, which means that significant changes in physicochemical properties occurred in CCF at 10% and 15% oxidative weight loss. When the oxidation weight loss rate was 10%, the compressive linear strength decreased to 32% of the original value.

The mechanical properties of ACCF have subtle differences compared to CCF. It can be reasonably inferred that its overall change trend is the same as that of CCF. Compared with CCF, the modulus of ACCF is about 3.8% higher, and the strength is about 6.9% higher, which may be because the SiC particles deposited on the carbon skeleton have a certain strengthening effect on the carbon skeleton.

### 3.3. Thermal Properties Analysis

**Figure 5:**
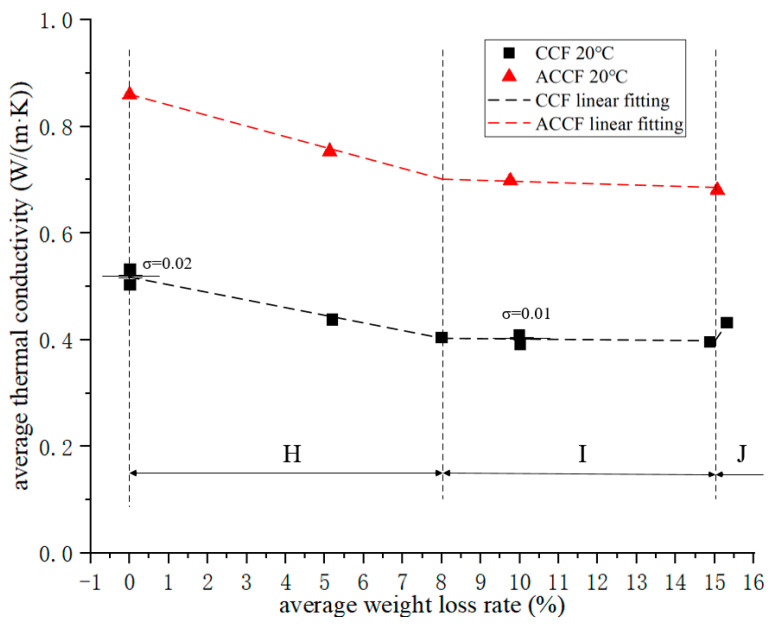
Average thermal conductivity–average weight loss rate curve.

The thermal performance of the specimen measured by Hot Disk is the average value of the two specimens, so when studying the relationship between the thermal properties of CCF and ACCF and the oxidation weight loss rate, the abscissa takes the arithmetic mean of the two specimens, namely the average oxidation weight loss rate, thus obtaining the average thermal conductivity–average oxidation weight loss rate curve of CCF and ACCF (as shown in Figure 5). Considering that the difference between the oxidation weight loss rates of the two specimens is relatively small, and the performance of the specimens must have dispersion, the method of studying the evolution law with the arithmetic mean value instead of the original value has sufficient precision.

Figure 5 shows that the thermal conductivity of CCF and ACCF decreased with the increase in the oxidation weight loss rate at 20 °C, and the lower the thermal conductivity of the material in the thermal protection structure, the safer the structure, which means that in the thermal protection structure there is no need to consider the attenuation of the thermal insulation performance of the structure when determining the life of the thermal protection structure [31,32]. The thermal conductivity decay curve of CCF is divided into three regions:

- In region H, the thermal conductivity decays linearly with the oxidation weight loss rate. This process continues until the CCF loses 8% of its weight. The thermal conductivity decreases by about 0.12 W/(m·K);
- In region I, the thermal conductivity also exhibits linear decay, but the decay slope decreases;
- In region J, the thermal conductivity suddenly increases. The mechanism is not yet clear, and further research is needed to eliminate interference.

Figure 5 shows that the thermal conductivity of ACCF is much larger than that of CCF, because the thermal conductivity of SiC particles deposited on the carbon skeleton is much larger than that of the carbon skeleton, and it still has the same trend as CCF. Due to the lack of sufficient data, it is impossible to determine whether ACCF will behave the same as CCF when the oxidative weight loss rate is greater than 15%.

### 3.4. SEM and Mechanism Analysis

**Figure 6:**
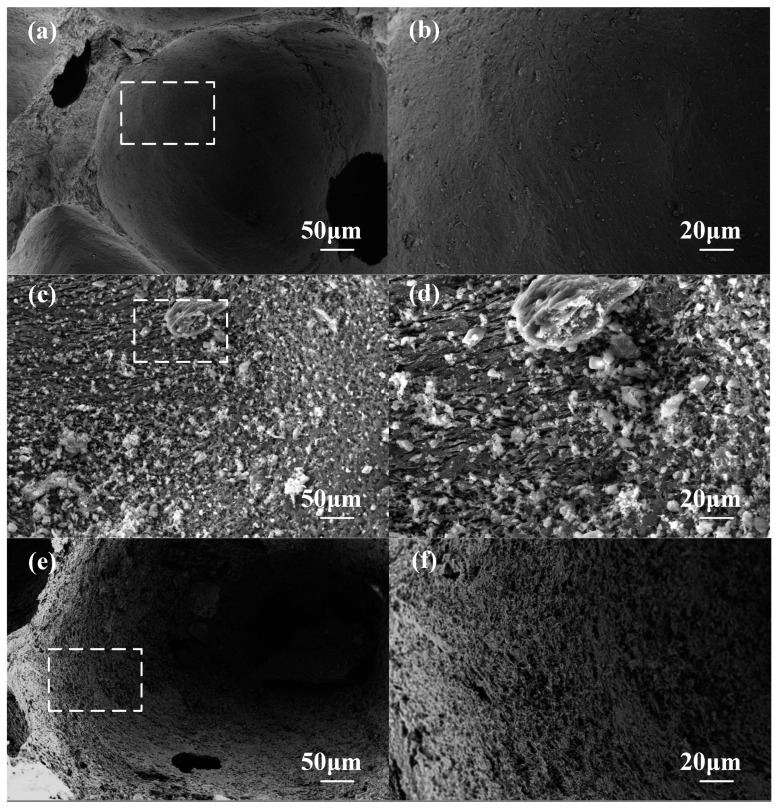
SEM images of pore wall surface: (**a**) low magnification of A-2-1, 5.24% weight loss; (**b**) high magnification of A-2-1, 5.24% weight loss; (**c**) low magnification of A-3-3, 9.39% weight loss; (**d**) high magnification of A-3-3, 9.39% weight loss; (**e**) low magnification of A-4-3, 14.56% weight loss; (**f**) high magnification of A-4-3, 14.56% weight loss.

**Figure 7:**
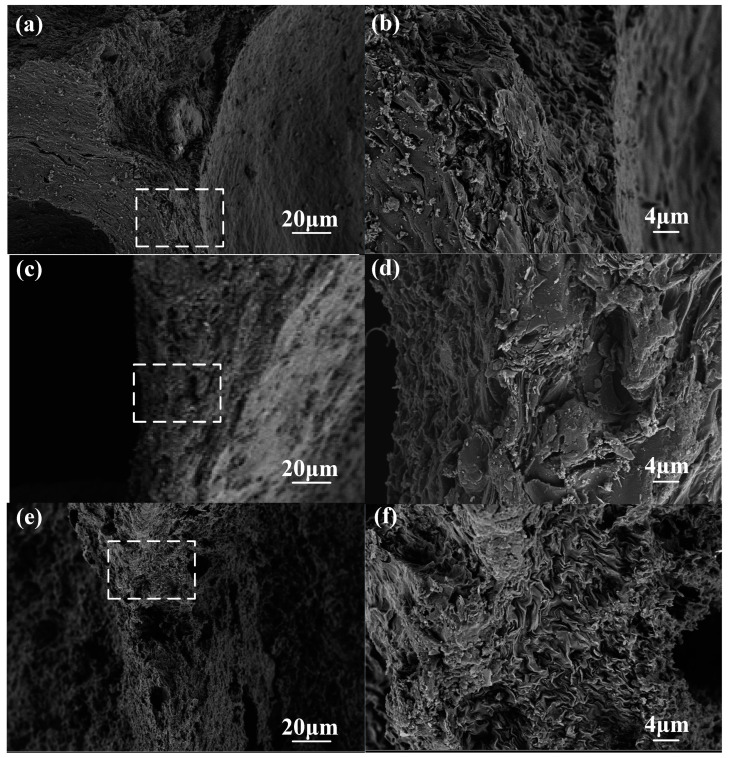
SEM images of pore-wall sections: (**a**) low magnification of A-2-1, 5.24% weight loss; (**b**) high magnification of A-2-1, 5.24% weight loss; (**c**) low magnification of A-3-3, 9.39% weight loss; (**d**) high magnification of A-3-3, 9.39% weight loss; (**e**) low magnification of A-4-3, 14.56% weight loss; (**f**) high magnification of A-4-3, 14.56% weight loss.

The SEM images of the pore wall surface are shown in Figure 6. The surface of the CCF pore wall with an oxidation weight loss rate of 5% (Figure 6a,b) is still dense and smooth, while the CCF pore wall surface with an oxidation weight loss rate of 14.56% (Figure 6e,f) has a porous and rough area of ablation. The SEM images of the whole wall section are shown in Figure 7. The pore walls of CCF with an oxidation weight loss rate of 5% (Figure 7a,b) are very dense, and irregular amorphous carbon is mixed with flake graphitized carbon, and the amorphous carbon is filled in the graphitized carbon intervals, with almost no crack visible. A large number of micron-scale pores appear on the inside and surface of the CCF pore wall with an oxidation weight loss rate of 14.56% (Figure 7e,f), and the sheet-like graphitized carbon is more obvious, occupying the majority in the field of view. This shows that as the degree of oxidation deepens, more amorphous carbon is consumed, and the remaining material is mostly graphitized carbon flakes. A reasonable explanation for the above phenomenon is that amorphous carbon is more prone to oxidation [23,24,25], which is due to the disorderly stacking of carbon atoms on its surface, which fails to form large-scale complete graphite crystallites, resulting in more surface defects and more oxygen-containing groups, which are more prone to oxidation reactions, while graphitized carbon is the opposite [25,26,27,28,29].

The fact that the amorphous carbon is more consumed and the relative content of graphitized carbon increases with the deepening of the oxidation degree observed in the SEM in Figure 6 and Figure 7 can well explain the transition of CCF from brittleness to plasticity.

The reason for the decrease in thermal conductivity was that the amorphous carbon was oxidized in large quantities, the pore walls changed from dense to porous, as shown in Figure 6, and solid heat transfer efficiency was reduced.

The average thermal conductivity–average weight loss rate curve has the same trend as the compressive properties–weight loss rate curve and the compressive linear strength decay curve, and the turning points of the decay slope change are also close: the thermal conductivity curve is at 8% and 15% weight loss rate, while the compression is 10% and 15%. It is clear that this trend is strongly related to the changing relative content of amorphous carbon and graphitized carbon during the CCF oxidation process, but its specific mechanism of action, whether there are other factors, and why the mechanical and thermal properties change after 15% still remain to be studied. These studies may provide solutions for improving the residual mechanical properties of materials after oxidation.

## 4. Conclusions

Antioxidation treatment has a small effect on the mechanical properties of CCF, but it will significantly increase the thermal conductivity of CCF, which has a negative impact on its thermal insulation performance. When designing thermal protection structures, attention must be paid to the measurement of thermal conductivity of ACCF and the SiC content. Antioxidation treatment did not alter the trend of change in the mechanical and thermal properties of CCF with the oxidative weight loss rate.

When the weight loss rate was less than 15%, the thermal conductivity of CCF decreased with the increase in weight loss rate; when the weight loss rate was greater than 15%, the thermal conductivity of CCF increased with the increase in oxidative weight loss rate. The compressive modulus and compressive linear strength of CCF respectively decreased to 40% and 32% of their initial value when the weight loss rate was 10%, and to 31% and 20% of their initial value when the weight loss rate was 15%. However, the mechanical properties of CCF decreased rapidly when the weight loss rate was above 15%. Considering that the compressive modulus and strength of CCF used in thermal protection structures have relatively high design margins, CCF can be used with the maximum weight loss rate of 15%.

The change in the mechanical properties of CCF with the weight loss rate is related to the relative content of amorphous carbon and graphitized carbon, and the mechanical behavior of CCF is similar to that of PCF when the degree of oxidation increases. This paper provides an important entry point for the study of the oxidation mechanism from a macroscopic point of view, and an important connection between the microscopic mechanism and macroscopic properties.

## Figures and Tables

**Table 1:** Experimental results.

Specimen Number	Initial Mass/g	Final Mass/g	Weight Loss Ratio	Module/MPa	Linear Strength/MPa	Average Weight Loss Ratio	Average Thermal Conductivity (20 °C) /(W/(m·K))
A-1-1	7.4213	7.4213	0.00%	916	9.19	0.00%	0.5341
A-1-2	7.4260	7.4260	0.00%	821	8.39		
A-1-3	6.0213	6.0213	0.00%	728	6.98	0.00%	0.5034
A-1-4	5.6688	5.6688	0.00%	765	7.86		
A-2-1	5.7217	5.4217	5.24%	572	5.03	5.18%	0.4391
A-2-2	6.0223	5.7138	5.12%	591	5.42		
A-2-3	5.9108	5.3801	8.98%	335	3.30	7.99%	0.4055
A-2-4	6.1611	5.7303	6.99%	472	4.73		
A-3-1	5.8492	5.2515	10.22%	320	2.29	9.98%	0.4106
A-3-2	5.5489	5.0087	9.74%	313	2.41		
A-3-3	5.8132	5.2673	9.39%	314	3.16	10.01%	0.3915
A-3-4	6.0056	5.3675	10.63%	309	2.61		
A-4-1	6.0228	5.1307	14.81%	248	2.25	14.86%	0.3966
A-4-2	6.1698	5.2499	14.91%	241	1.97		
A-4-3	6.0112	5.1358	14.56%	243	1.66	15.30%	0.4329
A-4-4	5.9501	4.9956	16.04%	92.4	0.91		
B-1-1	6.4214	6.4214	0.00%	864	8.55	0.00%	0.8599
B-1-2	6.4970	6.4970	0.00%	756	7.99		
B-2-1	6.7515	6.4206	4.90%	533	5.42	5.13%	0.7537
B-2-2	6.5522	6.2015	5.35%	591	5.90		
B-3-1	6.6258	5.9754	9.82%	350	3.44	9.76%	0.6988
B-3-2	6.4204	5.7975	9.70%	341	3.42		
B-4-1	6.6987	5.6996	14.91%	254	2.81	15.06%	0.6813
B-4-2	6.5103	5.5198	15.21%	262	2.33		

## Data Availability

Not applicable.
